# Orthodromic Reciprocating Tachycardia Relying on Aberrant Conduction: The Need for a Larger Circuit

**DOI:** 10.19102/icrm.2024.15023

**Published:** 2024-02-15

**Authors:** Wentao Gu, Nanqing Xiong, Jian Li, Xinping Luo

**Affiliations:** 1 Department of Cardiology, Huashan Hospital, Fudan University, Shanghai, China

**Keywords:** Aberrancy, accessory pathway, bundle branch block, orthodromic reciprocating tachycardia, supraventricular tachycardia.

## Abstract

Aberrant conduction during orthodromic reciprocating tachycardia (ORT) prolongs the ventriculoatrial conduction time, which can be essential for the maintenance of tachycardia in specific cases. We searched for ORT relying on aberrancy among 220 cases in our center. Three patients showed the phenomenon of aberrancy-dependent ORT. All accessory pathways were located at the lateral regions of the atrioventricular annulus. None of them had a baseline bundle branch block (BBB). Creating a functional BBB was necessary to induce the tachycardias. In two cases, termination of tachycardias was directly associated with resolution of the aberration. In the other case, re-entry required both BBB and slow pathway conduction. We conclude that extra transseptal time caused by aberrancy can be an integral part of the ORT circuit, which explains the infrequent and unsustainable episodes of ORT in certain patients and is useful in understanding the circuit and localizing the pathway.

## Introduction

During orthodromic reciprocating tachycardia (ORT), a functional bundle branch block (BBB) can be developed when the activation wavefront encroaches on the refractory period of the His–Purkinje system. The ventriculoatrial (VA) conduction time is shortened when BBB abates, with the bundle being ipsilateral to a concealed accessory pathway (AP).^1–3^ However, this may result in advancement of the wavefront to the refractory period of the circuit components, resulting in tachycardia termination. Here, we describe a group of patients sharing the phenomenon of ORT relying on aberrant conduction based on various mechanisms.

## Materials and methods

All patient studies with ORT undergoing an electrophysiology (EP) study in our center between January 2017 and January 2022 were reviewed. ORTs relying on aberrancy were selected using the following criteria: (1) multiple episodes of ORT with aberrant conduction, (2) the absence of a baseline BBB during sinus rhythm, (3) the absence of a narrow QRS form during tachycardia unless isoproterenol was used; and (4) restoration of bundle branch conduction giving rise to tachycardia termination or circuit alteration. During the procedure, a decapolar catheter was placed in the coronary sinus (CS) via the left femoral vein or right jugular vein. Two quadripolar catheters were usually inserted from the right femoral vein, one for the His bundle recording and the other positioned at the RV apex or high right atrium (HRA). The study was approved by the ethics committee of Huashan Hospital, Fudan University (approval no. KY2019-552, approval date: November 26, 2019). Informed consent was obtained from all subjects.

## Results

### Baseline information

Among 220 cases of ORT, 94 (42.7%) cases showed BBB during tachycardia, from which 3 (1.4%) patients fulfilled the study criteria. The baseline and EP characteristics of the cases are shown in **Table 1**.

### Case 1

A 56-year-old man with a history of palpitation and electrocardiogram (ECG) evidence of pre-excitation was referred for an EP study. All recorded ECGs during tachycardia showed a left BBB type. The maximal pre-excitation pattern was consistent with a left free wall AP. Retrograde conduction was mapped to the distal CS when pacing from the right ventricle (RV). Dual atrioventricular (AV) nodal physiology was not present. Tachycardia was induced by an atrial extrastimulus that was blocked in the AP, conducted over the AV node, and was associated with a left BBB.

**Figure 1:**
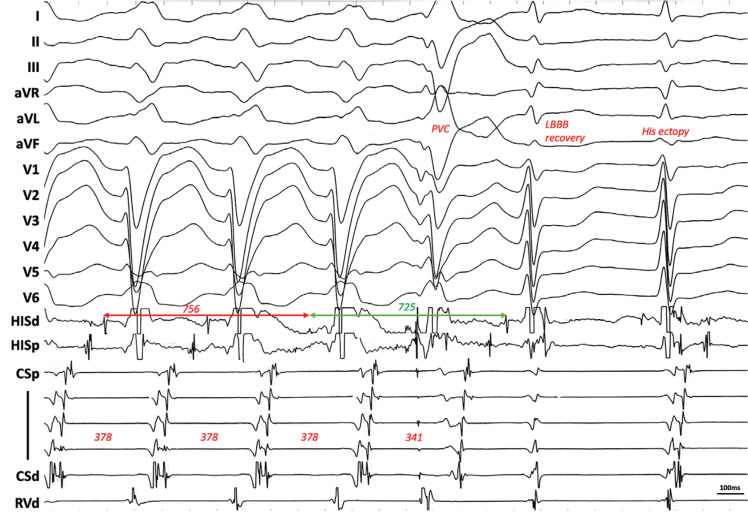
In case 1, a His-refractory premature ventricular contraction was delivered during tachycardia. The next A and subsequent His were advanced, after which the tachycardia was terminated with pathway block. The next beat was a catheter-induced ectopy followed by sinus rhythm. See text for discussion. *Abbreviations:* LBBB, left bundle branch block; PVC, premature ventricular contraction.

A late premature ventricular contraction (PVC) was delivered when the His bundle was committed, advanced the next A, and reset the tachycardia, which was diagnostic for ORT. Left bundle branch conduction was ultimately restored, which narrowed the next QRS complex. This finding is very suggestive of the perpetuation of BBB due to transseptal invasion. Tachycardia terminated when the wavefront reached the ventricular insertion site of the AP, which was refractory **(Figure 1)**. Mapping of the AP was performed using the transseptal approach during RV pacing. The earliest atrial activation site was located at the anterolateral mitral annulus (MA) with a sharp potential, where radiofrequency application successfully blocked the AP. The patient has been free from the tachycardia for 1 year.

### Case 2

A 64-year-old man presented with frequent episodes of tachycardia, which always terminated spontaneously. The only ECG from the emergency room showed a wide complex tachycardia with a left BBB. His tortuous femoral veins made catheterization difficult; therefore, only an RV catheter was inserted from the left femoral vein, with the CS catheter placed from the jugular vein for an EP study. The earliest atrial activation site was CS3–4 during RV pacing, indicating a left lateral AP. Atrial pacing and extrastimulus did not show pre-excitation, intraventricular block, or dual AV nodal physiology.

**Figure 2:**
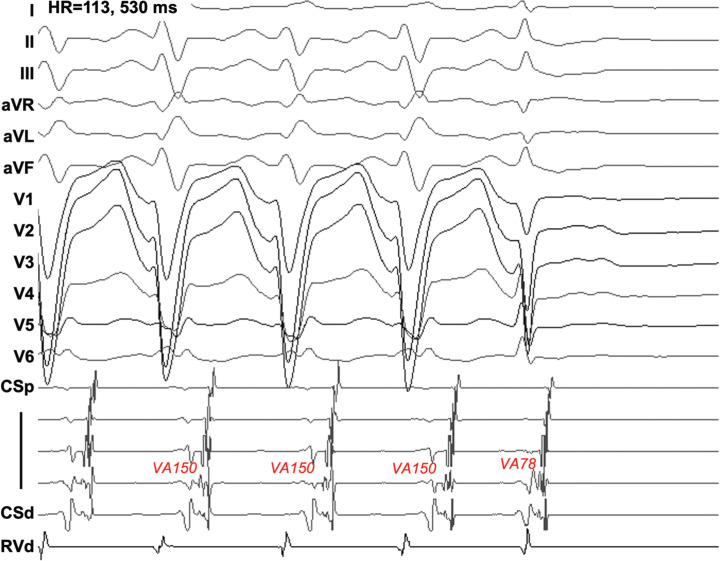
In case 2, orthodromic reciprocating tachycardia with aberrancy was terminated after spontaneous resolution of left bundle branch block, with advanced V and A at the mitral annulus. Note that the timing of the right ventricular apex was almost unchanged. *Abbreviation:* HR, heart rate.

The clinical tachycardia could only be induced by RV extrastimulus with isoproterenol, which lasted <10 beats, compatible with ORT with aberrancy. Termination of the tachycardia was always preceded by a narrow QRS complex, after advancement of the A, suggesting an anterograde block in the AV junction **(Figure 2)**. The pathway was ablated at the anterolateral MA using the retrograde approach, after which no tachycardia could be induced. The patient has been symptom-free for >3 years.

### Case 3

**Figure 3:**
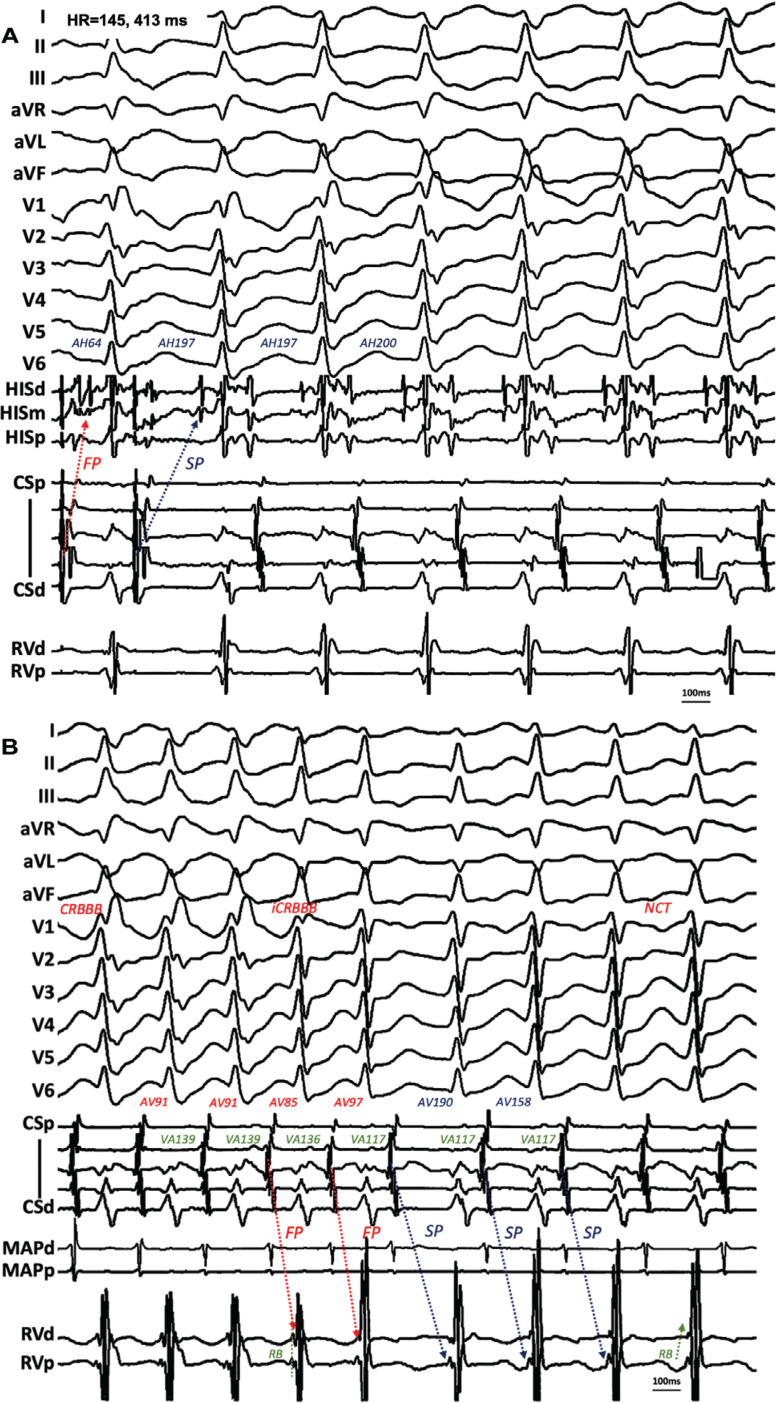
**A:** In case 3, the second beat conducting over the slow pathway with complete right bundle branch block (CRBBB) induced orthodromic reciprocating tachycardia, followed by A–H showing slow pathway perpetuation. **B:** Atrioventricular conduction was over the fast pathway in the first three beats, with the resolution of CRBBB in the next ones, followed by a shortened V–A interval and subsequent marked atrioventricular prolongation in the sixth beat. Note the subtle change in the right bundle conduction. See text for discussion. *Abbreviations:* AV, atrioventricular; CRBBB, complete right bundle branch block; iCRBBB, incomplete right bundle branch block; NCT, narrow complex tachycardia; SP, slow pathway; VA, ventriculoatrial.

A 24-year-old woman with ECG findings of pre-excitation presented with frequent but short episodes of palpitations. Extrastimuli from the HRA revealed maximal pre-excitation, suggesting a right anterolateral pathway and dual AV nodal conduction. Retrograde conduction over the AP was also confirmed. However, the tachycardia could not be initiated from either the RV or HRA. Stimulus from the left atrium, however, readily induced the tachycardia by conducting solely over the slow pathway of the AV node and causing a concomitant functional right bundle branch block (RBBB), with subsequent beats showing A–H intervals identical to the last paced beat, indicating repeated slow pathway conduction **(Figure 3A)**. A His-refractory PVC during tachycardia confirmed the mechanism of ORT. Thus, sustained tachycardia could only be initiated with RBBB and slow pathway conduction.

When isoproterenol (8 μg/min) was given during tachycardia, fast pathway and right bundle branch conduction were both improved, followed by a paradoxically longer tachycardia cycle length (TCL) owing to marked AV prolongation **(Figure 3B)**, indicating anterograde conduction over the slow pathway. The narrow tachycardia was neither sustainable nor re-inducible when high-dose isoproterenol was discontinued. The pathway was eliminated at 11 o’clock on the tricuspid annulus with the help of a deflectable sheath. After AP ablation, slow pathway conduction remained without echo beat or inducible AV nodal re-entrant tachycardia. The patient remained without symptoms at 2-year follow-up.

## Discussion

### Major findings

In this report, we demonstrate the role of aberrancy being essential for the maintenance of ORT. The major findings included:

1. In the cases of ORTs relying on aberrancy, all APs were ipsilateral of BBB
2. Tachycardia episodes were often brief
3. Stimulation during single bundle branch refractoriness is required for inducing tachycardia
4. Resolution of aberration was followed by the shortening of the subsequent V–A interval, which was responsible for tachycardia cessation
5. The block site of tachycardia after BBB resolution was either at the AP or AV node

### Basic mechanism for functional bundle branch block-dependent orthodromic reciprocating tachycardia

When aberration is present during ORTs mediated by APs located ipsilateral to the blocked bundle, the impulse travels down the contralateral bundle branch and lengthens the VA time.^1–3^ Meanwhile, the retrograde penetration into the blocked bundle branch is perpetuated by transseptal conduction during tachycardia, which can abate spontaneously or be restored by a ventricular extrastimulus peeling back the refractoriness of the bundle.^4^

As a macro–re-entrant tachycardia, ORT can be theoretically non-inducible in patients with APs if the conduction properties of the AP or AV node do not fulfill the prerequisite of re-entry. However, in the setting of aberrancy, the extra transseptal conduction time of 40 ms is added to VA conduction, which allows for wavefront arrival outside refractoriness of the circuit components.^2^ Conversely, the restoration of bundle branch conduction can shorten the intraventricular time and terminate tachycardia.

In our report, all APs were from the lateral regions of AV rings, which provided a more significant increase in TCL than the septal pathway (provided) when BBB occurred. To induce tachycardia in this setting, anterograde conduction should meet multiple requirements, including: (1) the AP is refractory, (2) the AV node is excitable, and (3) the BBB is ipsilateral to the pathways. Therefore, we show that the timing and site of pacing as well as the conduction properties of the AV node, AP, and His–Purkinje system may be involved in the initiation of aberrancy as well as tachycardia perpetuation.

### Electrophysiological findings supporting the need for aberrancy

**Figure 4:**
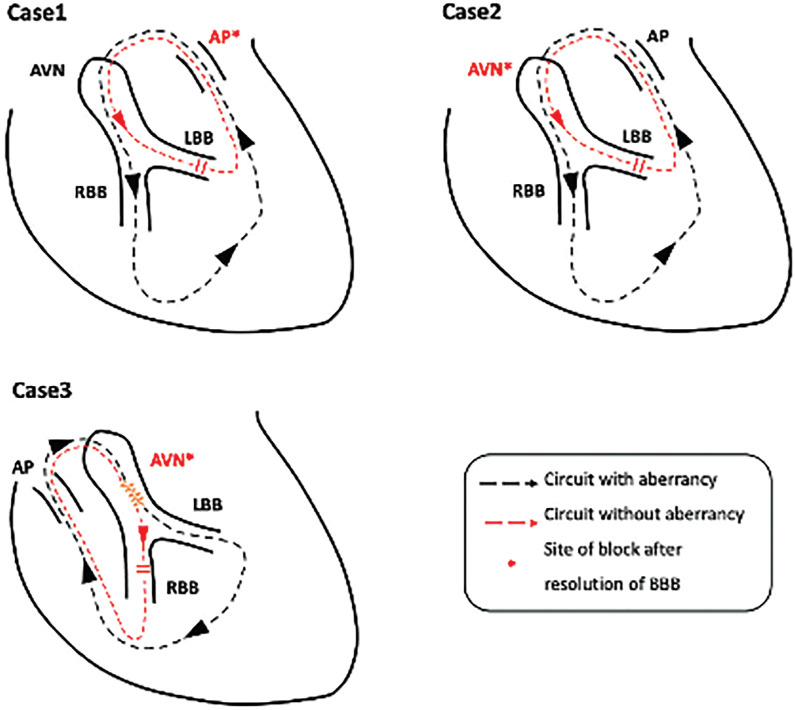
The three types of orthodromic reciprocating tachycardia circuit were demonstrated in the series, which could be terminated or altered with the resolution of aberrancy, including block at the accessory pathway, atrioventricular node, and slow pathway. *Abbreviations:* AVN, atrioventricular node; AP, accessory pathway; BBB, bundle branch block; LBB, left bundle branch; RBB, right bundle branch.

When bundle branch conduction was improved, the emerging wavefront encountered the refractory period of either the AP or AV conduction system and resulted in termination of the tachycardias **(Figure 4)**.

In case 1, a His-refractory PVC advanced the subsequent A and His through the AP. Meanwhile, it peeled back the refractoriness of the left bundle by “pre-exciting” it from the retrograde direction, which narrowed the next QRS. The aforementioned two effects advanced the ventricular signal at the anterolateral MA (V_CS1–2_) by 60 ms, which was in the refractory period of AP. Case 2 had a similar mechanism as case 1, while the resolution of the blockage was spontaneous. The local V and following A on the left side were advanced in this beat, which made the wavefront reach the AV junction (could be the AV node or His–Purkinje system) during refractoriness.

In case 3, stable tachycardia could only be induced when RBBB was created when associated with slow pathway conduction. With isoproterenol, the slow pathway was replaced by a fast pathway to participate in re-entry, followed by improvement of the aberration, resulting in the premature depolarization of the AP, which afterward advanced the input into the AV junction and again blocked the fast pathway. This suggested that both the infra-His block and slow pathway conduction contributed to maintaining re-entrant activation. Interestingly, the narrow QRS tachycardia had a longer TCL than the wide complex owing to a marked increase in the A–V interval, demonstrating a finding in opposition to “Coumel’s law.”^5^

### Proportion of orthodromic reciprocating tachycardias relying on aberrancy

Among our 95 cases of ORT with aberrancy, only three showed solid evidence proving that BBB was indispensable. The other cases included ORT cases with pre-existing BBB, cases with concomitant wide and narrow QRS complexes, and those with only wide QRS complexes. In the latter situation, sensed extrastimuli have been attempted but failed to reactivate the blocked bundle. Therefore, it remains unknown whether those cases only showing wide QRS were relying on an aberration or not. Additionally, the conduction velocity and refractory period of the AV node and AP can be altered by medications and autonomic nerve activity, which may influence the dependence of aberrancy during EP study.

### Limitations

We enrolled a small number of cases in this study due to the rare presentation of the observed phenomenon. In addition, episodes of tachycardia in case 2 were too short to allow for multiple differential diagnostic maneuvers.

## Conclusions

In ORTs using free wall APs, increased transseptal conduction time caused by BBB can be an integral part of the circuit, enabling re-entrant activation by enlarging the circuit. This explains the infrequent or late onset and unsustainable episodes of ORT in certain patients with APs, which can also be regarded as a useful tool in understanding the mechanism and localizing the AP.

## Figures and Tables

**Table 1:** Baseline Data and Electrophysiological Characteristics

	Case 1	Case 2	Case 3
Sex	Male	Male	Female
Age (years)	56	75	24
LVEF (%)	69	68	65
AP location	Anterolateral MA	Anterolateral MA	Anterolateral TA
AP conduction property	Anterograde and retrograde	Retrograde	Anterograde and retrograde
AVN ERP (ms)	230	280	260 (SP)
AVN RRP (ms)	470	430	380
AP anterograde ERP (ms)	460	—	300
AP retrograde ERP (ms)	380	320	280

*Abbreviations:* AP, accessory pathway; AVN, atrioventricular node; ERP, effective refractory period; LVEF, left ventricular ejection fraction; MA, mitral annulus; RRP, relative refractory period; SP, slow pathway; TA, tricuspid annulus. Refractory periods were measured by using extrastimulus at baseline or after AP ablation, without isoproterenol given.
